# Two new species of the genus *Indolipa* Emeljanov (Hemiptera, Fulgoromorpha, Cixiidae) from Yunnan Province, China, with a key to species

**DOI:** 10.3897/zookeys.956.51326

**Published:** 2020-08-06

**Authors:** Yan Zhi, Pei Zhang, Lin Yang, Xiang-Sheng Chen

**Affiliations:** 1 Institute of Entomology, Guizhou University, Guiyang, Guizhou, 550025, China; 2 Laboratory Animal Center, Guizhou Medical University, Guiyang, Guizhou 550025, China; 3 The Provincial Special Key Laboratory for Development and Utilization of Insect Resources of Guizhou, Guizhou University, Guiyang, Guizhou, 550025, China; 4 Xingyi Normal University for Nationalities, Xingyi, Guizhou, 562400, China

**Keywords:** Fulgoroidea, morphology, Oriental region, planthopper, taxonomy

## Abstract

Two new species of *Indolipa* Emeljanov, 2001 (Fulgoromorpha, Cixiidae) from Yunnan Province, China, *I. fugongensis* Zhi & Chen, **sp. nov.** and *I. longlingensis* Zhi & Chen, **sp. nov.** are described. Color images for the adults of the two new species and line drawings for the genitalia are provided. In addition, a key to all known species of *Indolipa* Emeljanov is included.

## Introduction

The planthopper genus *Indolipa* was established by Emeljanov (2001) for sixteen species (previously in *Oliarus* Stål, 1862) in the tribe Pentastirini (Hemiptera, Cixiidae, Cixiinae), with *Oliarus indiensis* Van Stalle, 1991 as the type species. Recently, *Indolipa* has been studied taxonomically by Guo and Feng (2010) and Luo et al. (2019), with three Chinese species published successively. Thus, this genus so far includes nineteen valid species in total, and all its fauna is distributed in the Oriental and Palaearctic regions (Bourgoin 2020). Previously five species in this genus have been recorded from China: *I. fopingensis* Luo, Liu & Feng, *I. gansuensis* Feng, *I. huapingensis* Luo, Liu & Feng, *I. kurseongensis* (Distant) and *I. tappanus* (Matsumura).

Recent efforts in studying specimens collected from China revealed two new species, *I. fugongensis* Zhi & Chen, sp. nov. and *I. longlingensis* Zhi & Chen, sp. nov. Hence, the species number of *Indolipa* has been raised to twenty-one, with seven species occurring in China.

## Materials and methods

The morphological terminology and measurements follow Bourgoin (1987), Bourgoin (1993) and Bourgoin et al. (2015) respectively for male genitalia, female genitalia and wing venation. Body length was measured from apex of vertex to tip of forewing; vertex length was measured in the median length of vertex (from apical transverse carina to tip of basal emargination). Fuchsin staining was used to highlight female genitalia structures studied. External morphology and drawings were done with the aid of a Leica MZ 12.5 stereomicroscope. Photographs were taken with KEYENCE VHX-1000 system. Illustrations were scanned with CanoScan LiDE 200 and imported into Adobe Photoshop 7.0 for labeling and plate composition. The distribution map was generated with ARCGIS 10.5. The dissected male and female genitalia are preserved in glycerin in small plastic tubes pinned together with the specimens.

The type specimens examined are deposited in the Institute of Entomology, Guizhou University, Guiyang, Guizhou Province, China (GUGC).

## Taxonomy

### Indolipa

Taxon classificationAnimaliaHemipteraCixiidae

Emeljanov, 2001

20CE285A-5E3F-5689-8FB6-492C9E7851AF

Indolipa Emeljanov, 2001: 72; Guo and Feng 2010: 34; Luo et al. 2019: 185.

#### Type species.

*Oliarus indiensis* Van Stalle, 1991, by original designation.

#### Diagnosis.

See Luo et al. (2019: 185).

#### Distribution.

China (Gansu, Guangxi, Hubei, Hunan, Shaanxi, Tibet, Taiwan, Yunnan), India, Indonesia (Borneo), Malaysia, Myanmar, Singapore, Sri Lanka.

### Key to species of the genus *Indolipa* Emeljanov

**Table:** 

1	Vertex with subapical transverse carina connected to apical border by two longitudinal distinct carinae or two indistinct elevations (Figs 1C, 3C)	**2**
–	Vertex with subapical transverse carina not connected with apical border	**20**
2	Vertex broader than long (Figs 1C, 3C)	**3**
–	Vertex longer than or equally long as broad	**7**
3	Vertex without median carina (Fig. 3C)	**4**
–	Vertex with median carina (Fig. 1C)	**5**
4	Right side of endosoma with one ribbon-like process, with two short laminal processes on the process basally (Fig. 3K)	***I. longlingensis* sp. nov.**
–	Right side of endosoma with two ribbon-like processes, without processes on the process basally (Luo et al. 2019: fig. 17)	***I. fopingensis***
5	Forewing with 10 apical cells (Fig. 1E), chaetotaxy of hind tarsi: 7/7	***I. fugongensis* sp. nov.**
–	Forewing with 12 apical cells, chaetotaxy of hind tarsi: 7/5	**6**
6	Tegmina with one complete and nearly straight transverse fuscous fascia in basal area; female without an incision on the caudal margin of the pregenital sternite	***I. fusconebulosus***
–	Tegmina with two narrow spots and three somewhat long curved linear spots in basal area; female with an incision on the caudal margin of the pregenital sternite	***I. binghami***
7	Anal segment symmetrical	**8**
–	Anal segment asymmetrical	**14**
8	Periandrium of aedeagus without process (Van Stalle 1991: fig. 334)	***I. lawitensis***
–	Periandrium of aedeagus with process(es)	**9**
9	Periandrium of aedeagus with a bifurcate process	**10**
–	Periandrium of aedeagus without bifurcate process	**12**
10	Bifurcate process of periandrium on its dorsal margin (Van Stalle 1991: fig. 411)	***I. bidiensis***
–	Bifurcate process of periandrium on its ventral margin	**11**
11	Endosoma with four spinose processes basally (Van Stalle 1991: figs 347, 348); forewing with 10 apical cells; chaetotaxy of hind tarsi: 7/5–6	***I. madrasensis***
–	Endosoma with three spinose processes basally (Van Stalle 1991: figs 354, 355); forewing with 11 apical cells; chaetotaxy of hind tarsi: 7–9/7	***I. nilgiriensis***
12	Vertex 1.5 times as long as broad; periandrium of aedeagus with five spinose processes apically, endosoma curved in a semi-circle, and three spinose processes on its dorsal margin (Van Stalle 1991: fig. 341)	***I. sabahensis***
–	Vertex as long as broad; periandrium and endosoma of aedeagus without features as above	**13**
13	Mesonotum black with two yellow fasciae between outer carinae; periandrium of aedeagus with two spinose processes on left side, endosoma with five processes (Van Stalle 1991: figs 369, 370)	***I. indiensis***
–	Mesonotum entirely black; periandrium of aedeagus with one spinose process on left side, endosoma with four processes (Van Stalle 1991: figs 377, 378)	***I. greeni***
14	Aedeagus with ventral margin of periandrium without laminal process; forewing with 12 apical cells	**15**
–	Aedeagus with ventral margin of periandrium with a laminal process basally; forewing with 9–10 apical cells	**17**
15	Ventral margin of periandrium with a spinose process near apex (Van Stalle 1991: fig. 328)	***I. pahangensis***
–	Ventral margin of periandrium without process	**16**
16	Pygofer with left lateral margin rounded at apex; left side of periandrium with a spinose process (Van Stalle 1991: figs 311–313)	***I. malayensis***
–	Pygofer with left lateral margin slightly incised at apex; periandrium without process (Van Stalle 1991: figs 320–322)	***I. tamangensis***
17	Left side of endosoma with a circle process (Tsaur et al. 1988: fig. 6C)	***I. tappanus***
–	Left side of endosoma without circle process	**18**
18	Left side of endosoma with a bifurcate process	**19**
–	Left side of endosoma without bifurcate process (Luo et al. 2019: fig. 27)	***I. huapingensis***
19	Right side of endosoma with two long subparallel ribbon-like processes (Guo and Feng 2010: fig. 9)	***I. gansuensis***
–	Right side of endosoma with one produced rod-like process	***I. kurseongensis***
20	Vertex with subapical carina almost straight, median carina absent (Van Stalle 1991: Fig. 365); pronotum black; chaetotaxy of hind tarsi: 7–8/7	***I. thekkadiensis***
–	Vertex with subapical carina angulate, median carina present (Van Stalle 1991: fig. 409); pronotum yellow; chaetotaxy of hind tarsi: 6/5	***I. brunnifrons***

### Indolipa fugongensis

Taxon classificationAnimaliaHemipteraCixiidae

Zhi & Chen sp. nov.

F5D3F9A5-CB5C-53A9-90B8-7B4A6174C4C8

http://zoobank.org/E1D648F6-C5E8-4A0B-B780-674920F8924E

Figures 1A–N , 2A–H

#### Type material.

***Holotype***: ♂, China: Yunnan Province, Fugong County (26°54'N, 98°52'E), 17–18 May 2010, Pei Zhang, Yan-Li Zheng and Yi Yan. ***Paratypes***: 7♂♂6♀♀, same data as holotype.

#### Description.

Body length: male 4.9–6.2 mm (*N* = 8), female 6.0–6.8 mm (*N* = 6).

**Figure 1.**
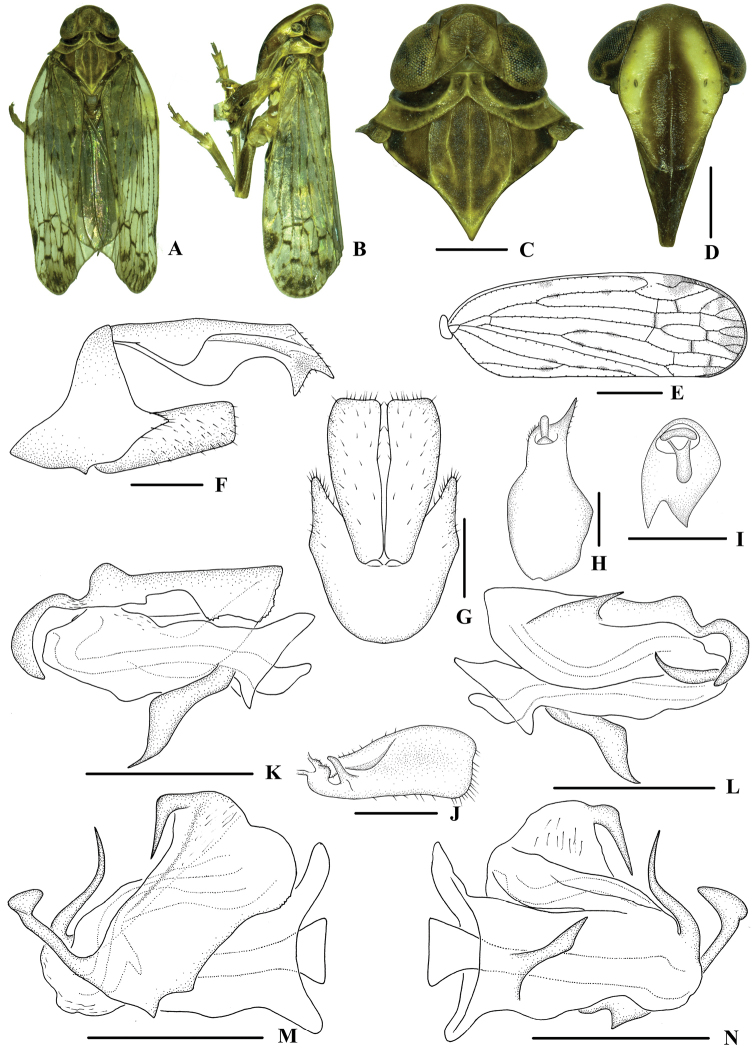
*Indolipa fugongensis* sp. nov., male **A** habitus, dorsal view **B** habitus, lateral view **C** head and thorax, dorsal view **D** face, ventral view **E** forewing **F** genitalia, lateral view **G** pygofer and gonostyli, ventral view **H** anal segment, dorsal view **I** anal segment, caudal view **J** gonostyli, inner lateral view **K** aedeagus, right side **L** aedeagus, left side **M** aedeagus, dorsal view **N** aedeagus, ventral view. Scale bars: 0.5 mm (**C–D**, **F–N**); 1.0 mm (**E**).

***Coloration*.** General color dark brown (Fig. 1A–D). Eyes dark brown, ocelli yellow. Vertex dark brown. Face generally brown, margins yellow. Rostrum pale brown. Pronotum and mesonotum dark brown, carinae paler. Forewing semi-translucent, brown (sometimes blackish brown), stigma dark brown, apex of forewing with several small blackish brown spots, veins generally brown with discontinuous blackish brown markings. Hind tibiae pale brown and abdominal sternites blackish brown.

***Head and thorax*.** Vertex (Fig. 1A, C) broad, 1.3 times wider than long; anterior margin arched convex; subapical transverse carina arc-shaped, connected with anterior border of vertex by two longitudinal small carinae; median carina only discernible at basal half; posterior margin nearly excavated at right angle. Frons (Fig. 1D) 1.6 times as wide as long, with median carina distinct and fork of median carina near apex. Pronotum (Fig. 1C) 1.1 times longer than vertex, posterior margin concaved in right angle. Mesonotum 1.1 times longer than pronotum and vertex combined. Forewing (Fig. 1E) 3.0 times longer than wide, with 10 apical and 5 subapical cells; fork Sc+RP basad of fork CuA1+CuA2; first crossvein r-m basad of fork MP; RP 3 branches, MP with 4 terminals: MP 1, MP2, MP3, and MP4, fork MP1+MP2 basad of fork MP3+MP4. Hind tibia with 5 lateral spines; chaetotaxy of hind tarsi: 7/7, second segment of hind tarsus without platellae.

***Male genitalia*.** Pygofer (Fig. 1F, G) symmetrical, dorsal margin concave and U-shaped ventrally, widened towards apex and slightly convex in the middle; in lateral view, lateral lobes triangularly extended caudally. Medioventral process absent, replaced by two small projections. Anal segment (Fig. 1F, H, I) asymmetrical, in lateral view, dorsal margin almost straight, ventral margin convex in the middle, right lobe larger than left one and apical lobe extended ventrally; 2.2 times longer than wide in dorsal view; anal style finger-like, beyond anal segment. Gonostyli (Fig. 1F, G, J) symmetrical in ventral view; in inner lateral view, trapezoidal, apical margin transverse, base with a deep round excavation and a tusk-like tooth. Aedeagus (Fig. 1K–N) with total of four processes. Base of periandrium with a curved laminal process positioning slightly to right side of its ventral margin, apex acute, directed ventrocaudally. Endosoma broad, convoluted with two sinuations, a right lateral one (Fig. 1K) and a left lateral one (Fig. 1L). In the right lateral view, a large laminal structure with a ribbon-like process apically, directed left-ventrocephalically. In left lateral view, the base of endosoma with a spinose process, apex directed left-dorsocephalically; a spinose process arising from apical 1/3 of endosoma on the dorsal margin, apex directed right-caudally.

**Figure 2.**
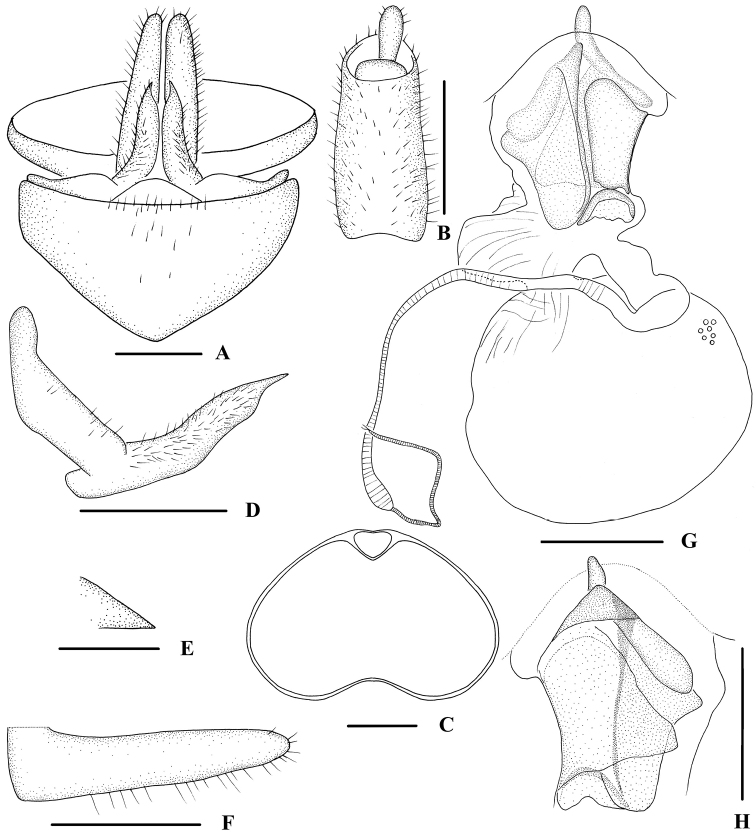
*Indolipa fugongensis* sp. nov., female. **A** genitalia, ventral view **B** anal segment, dorsal view **C** tergite IX, caudal view **D** gonapophysis VIII and gonocoxa VIII, ventral view **E** gonapophysis IX, ventral view **F** gonoplac, ventral view **G** posterior vagina and internal genitalia, ventral view **H** posterior vagina, dorsal view. Scale bars: 0.5 mm (**A–D, F–H**); 0.2 mm (**E**).

***Female genitalia*.** Pregenital sternite (Fig. 2A) with caudal margin slightly recessed, twice wider than long. Tergite IX (Fig. 2A, C) moderately sclerotized, with a large nearly oval wax plate. Anal segment (Fig. 2B) nearly rectangular, 2.2 times longer than wide in dorsal view, anal style finger-like. Gonapophysis VIII (Fig. 2D) reduced, apex acute. Gonapophysis IX (Fig. 2E) extremely short, triangular. Gonoplac (Fig. 2F) strap-shaped. Posterior vagina as shown in Fig. 2G, H. In ventral view, left side with a nearly triangular sclerite, which with a triangular process at the base; right side with a large sclerite bent towards the dorsal surface and a small semicircular sclerite near terminal. In dorsal view, basal area with a process and an oblong sclerite, which with a triangular pouch-like structure basally.

#### Distribution.

China (Yunnan) (Fig. 5).

#### Etymology.

The species name is derived from Fugong County, Yunan Province, where the type locality is located.

#### Remarks.

Male genitalia of *I. fugongensis* sp. nov. is similar to *I. kurseongensis* (Distant, 1911), but differs in: (1) the laminal process on the ventral margin of periandrium acute apically (in *I. kurseongensis*, the laminal process in the same position expanded apically); (2) in the right lateral view, base of endosoma without process (the latter with three processes); (3) left side of endosoma with two spinose processes (in *I. kurseongensis*, left side of endosoma with a S-shaped process and a Y-shaped process).

### Indolipa longlingensis

Taxon classificationAnimaliaHemipteraCixiidae

Zhi & Chen sp. nov.

8B90683A-5D6F-5867-995B-144CA3B38B49

http://zoobank.org/17EE8128-B900-4BB3-8511-B6CACC988A76

Figures 3A–N , 4A–H

#### Type material.

***Holotype***: ♂, China: Yunnan Province, Longling County (24°35'N, 98°41'E), 9–11 June 2011, Jian-Kun Long. ***Paratypes***: 22♂♂25♀♀, same data as holotype, Yu-Jian Li, Zai-Hua Yang and Jian-Kun Long.

#### Description.

Body length: male 5.3–5.8 mm (*N* = 23), female 6.2–6.7 mm (*N* = 25).

**Figure 3.**
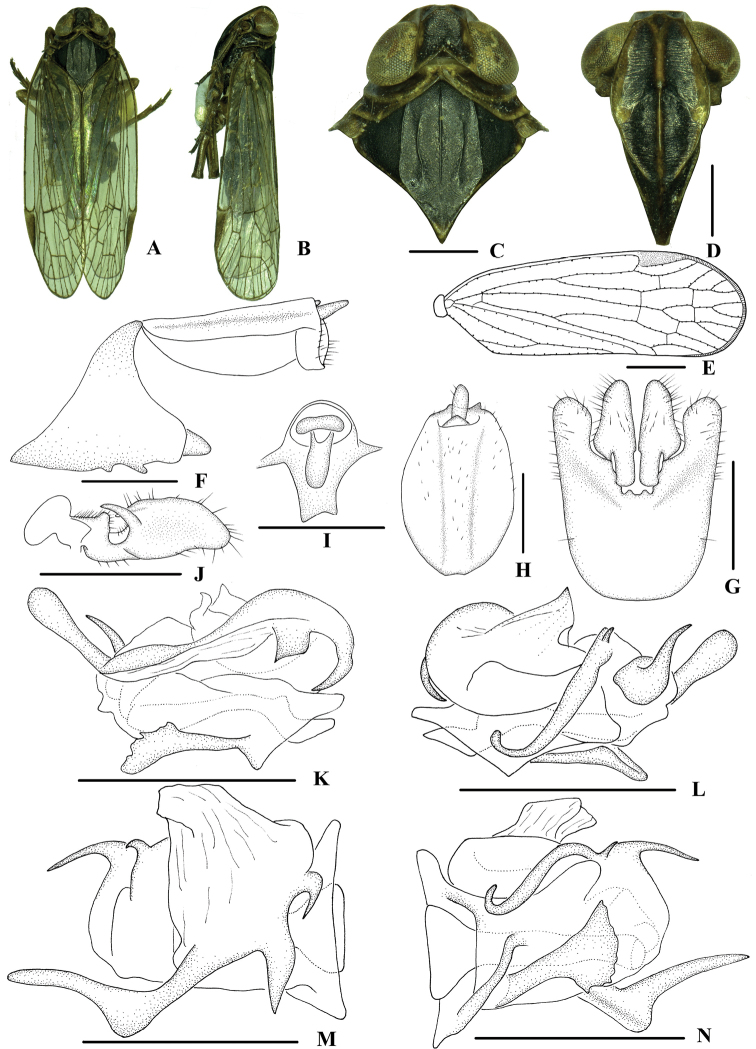
*Indolipa longlingensis* sp. nov., male **A** habitus, dorsal view **B** habitus, lateral view **C** head and thorax, dorsal view **D** face, ventral view **E** forewing **F** genitalia, lateral view **G** pygofer and gonostyli, ventral view **H** anal segment, dorsal view **I** anal segment, caudal view **J** gonostyli, inner lateral view **K** aedeagus, right side **L** aedeagus, left side **M** aedeagus, dorsal view **N** aedeagus, ventral view. Scale bars: 0.5 mm (**C–D, F–N**); 1.0 mm (**E**).

***Coloration*.** General color black (Fig. 3A–D). Eyes brown, ocelli yellowish brown. Vertex black. Face generally blackish brown, carinae and margins brown. Rostrum brown. Pronotum dark to blackish brown, carinae paler; mesonotum black. Forewing semi-translucent, pale brown, stigma brown. Hind tibiae and abdominal sternites blackish brown.

***Head and thorax*.** Vertex (Fig. 3A, C) broad, 1.3 times wider than long; anterior margin arched convex; subapical transverse carina arc-shaped, connected with anterior border of vertex by two longitudinal small carinae; median carina absent; posterior margin nearly excavated at right angle. Frons (Fig. 3D) 1.3 times as wide as long, with median carina distinct and fork of median carina near apex. Pronotum (Fig. 3C) 1.3 times longer than vertex, posterior margin concaved in obtuse angle. Mesonotum 1.5 times longer than pronotum and vertex combined. Forewing (Fig. 3E) 3.0 times longer than wide, with 10 apical and 5 subapical cells; fork Sc+RP slightly distad of fork CuA1+CuA2; first crossvein r-m basad of fork MP; RP 3 branches, MP with 4 terminals: MP 1, MP2, MP3, and MP4, fork MP1+MP2 distad of fork MP3+MP4. Hind tibia with 3 lateral spines; chaetotaxy of hind tarsi: 6/5, second segment of hind tarsus without platellae.

***Male genitalia*.** Pygofer (Fig. 3F, G) symmetrical, dorsal margin concave and U-shaped ventrally, widened towards apex; in lateral view, lateral lobes trapezoidally extended caudally. Medioventral process absent, replaced by two small projections. Anal segment (Fig. 3F, H, I) asymmetrical, in lateral view, dorsal margin almost straight, ventral margin convex, right lobe larger than left one and apical lobe extended ventrally; 1.5 times longer than wide in dorsal view; anal style finger-like, beyond anal segment. Gonostyli (Fig. 3F, G, J) symmetrical in ventral view; in inner lateral view, thumb-shaped, apical margin round, basal 1/3 with a deep round excavation and a tusk-like tooth. Aedeagus (Fig. 3K–N) with total of seven processes. Base of periandrium with a scoop-like laminal process positioning slightly to right side of its ventral margin, directed cephalad. Endosoma convoluted with two sinuations, a right lateral one (Fig. 3K) and a left lateral one (Fig. 3L). In the right lateral view, endosoma with a long ribbon-like process, apex slightly expanded and round, curving left-dorsocaudally; basal portion of the ribbon-like process with two short laminal processes, apex acute, directed ventrocaudally. In left lateral view, the base of endosoma with a strongly curved process, apex acute, directed dorsocaudally; a long rod-like process arising from basal 1/3 of endosoma on the dorsal margin, curving downwards, apex round, directed dorsally, base of the long process with an extremely short spinose process, apex directed dorsocaudally.

**Figure 4.**
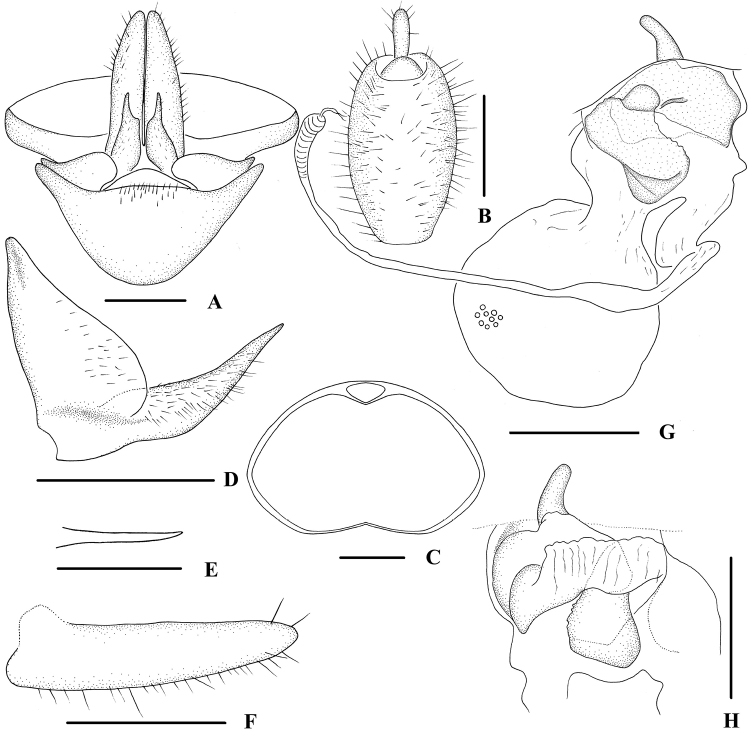
*Indolipa longlingensis* sp. nov., female. **A** Genitalia, ventral view **B** anal segment, dorsal view **C** tergite IX, caudal view **D** gonapophysis VIII and gonocoxa VIII, ventral view **E** gonapophysis IX, ventral view **F** gonoplac, ventral view **G** posterior vagina and internal genitalia, ventral view **H** posterior vagina, dorsal view. Scale bars: 0.5 mm.

***Female genitalia*.** Pregenital sternite (Fig. 4A) with caudal margin slightly convex in the middle, 2.3 times wider than long. Tergite IX (Fig. 4A, C) moderately sclerotized, with a large nearly oval wax plate. Anal segment (Fig. 4B) oval, 1.8 times longer than wide in dorsal view, anal style finger-like. Gonapophysis VIII (Fig. 4D) reduced, apex acute. Gonapophysis IX (Fig. 4E) comparatively short and thin. Gonoplac (Fig. 4F) strap-shaped. Posterior vagina as shown in Fig. 4G, H. In ventral view, left side with a nearly rectangular sclerite, which with a pouch-like structure at the base and terminal; in dorsal view, basal area with an irregular large sclerite, which with a process basally.

#### Distribution.

**Figure 5.**
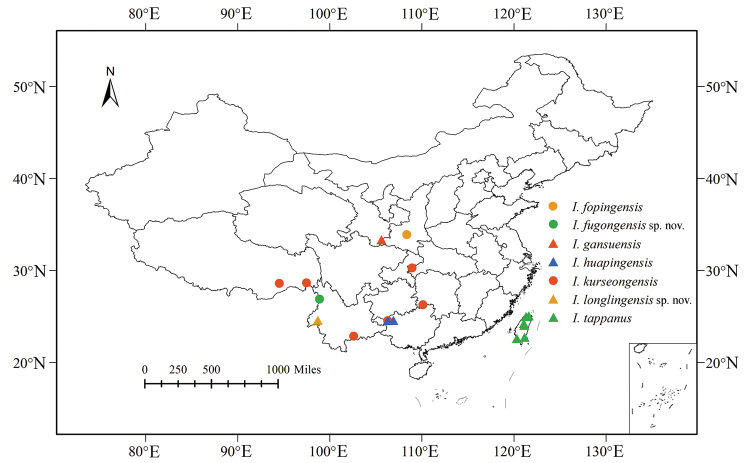
Distribution records of Chinese species of the genus *Indolipa*.

China (Yunnan) (Fig. 5).

#### Etymology.

The species name is derived from Longling County, Yunan Province, where the type locality is located.

#### Remarks.

Male genitalia of *I. longlingensis* sp. nov. is similar to *I. huapingensis* Luo, Liu & Feng, 2019, but differs in: (1) left side of endosoma with a long rod-like process at basal 1/3, which with an extremely short spinose process basally (the latter in the same position with a foliaceous process, which without spinose process basally); (2) ventral margin of endosoma without process (in *I. huapingensis*, ventral margin of endosoma with a tusk-like process); (3) forewing with 10 apical cells (the latter with 9 apical cells).

## Discussion

The Chinese species *Indolipa fopingensis*, *I. fugongensis* sp. nov., *I. gansuensis*, *I. huapingensis*, *I. kurseongensis*, *I. longlingensis* sp. nov. and *I. tappanus* share a similar screw-shaped aedeagus, and a similar basiventral process on the periandrium. We therefore believe that these species may be closely related. Based on the complex and variable geomorphological environment and rich biological resources in China, we expect that further new collections will increase the number of new records or species.

## Supplementary Material

XML Treatment for Indolipa

XML Treatment for Indolipa fugongensis

XML Treatment for Indolipa longlingensis
